# Quantification of individual proteins in silicone hydrogel contact lens deposits

**Published:** 2013-02-18

**Authors:** Negar Babaei Omali, Zhenjun Zhao, Hua Zhu, Daniel Tilia, Mark D.P. Willcox

**Affiliations:** 1 Brien Holden Vision Institute, Sydney, Australia; 2 School of Optometry and Vision Science, University of New South Wales, Sydney, Australia; 3 Australian School of Advanced Medicine, Macquarie University, Sydney, Australia

## Abstract

**Purpose:**

The aim of this study was to quantify specific proteins deposited on daily wear silicone hydrogel lenses used in combination with multipurpose disinfecting solutions (MPDSs) by applying multiple-reaction-monitoring mass spectrometry (MRM-MS).

**Methods:**

Balafilcon A or senofilcon A contact lenses used with different MPDSs on a daily wear schedule were collected. Each worn lens was extracted and then digested with trypsin. MRM-MS was applied to quantify the amounts of lysozyme, lactoferrin, lipocalin-1, proline-rich protein-4, and keratin-1 in the extracts.

**Results:**

The amount of protein extracted from the contact lenses was affected by the individual wearers, lens material, and type of care system used. Higher amounts of proteins were extracted from lenses after wear when they were used with an MPDS containing polyhexamethylene biguanide (PHMB) and poloxamer 407 compared with MPDSs containing polyquaternium-1 (PQ-1)/alexidine dihydrochloride with Tetronic 904 or PQ-1/ PHMB with poloxamine and sulfobetaine (p<0.05). There was a correlation between the amount of lipocalin-1 or keratin-1 extracted from lenses and symptoms of ocular dryness.

**Conclusions:**

The MRM-MS technique is a promising approach that could be used to reveal associations of individual proteins deposited on lenses with performance of contact lenses during wear.

## Introduction

Proteins [1-6], lipids [2,7,8], and mucins [9,10] are tear components that accumulate on contact lenses during wear. Proteins often reported as being detected in worn lens extracts include lysozyme, lactoferrin, lipocalin, proline-rich protein-4, and keratins [11-14], most of which, except certain keratins, are tear proteins. The major tear proteins include lysozyme, lactoferrin, secretory immunoglobulin A, and lipocalin [15-20]. Lysozyme, lipocalin, proline-rich protein-4, and lactoferrin with molecular masses of 14, 21–25, 15–21, and 78 kDa, respectively, are derived from the acinar cells of lacrimal glands [17,19,21-23]. Contact lens handling by contact lens wearers might contribute to contamination with keratins, as might interaction with the corneal and conjunctival epithelia [24], most likely through the mechanical force of a contact lens moving with every blink. There is evidence that protein accumulation and poor lens cleaning can contribute to clinical complications such as discomfort [3,25,26], reduced vision [27], infection [28,29], and inflammatory responses [30,31]. Increased deposits on contact lenses have been associated with increased corneal staining with fluorescein [25]. Daily wear of senofilcon A lenses has been associated with tear instability, decreased *MUC5AC* expression, and increased interleukin-6 in tears [32]. The type of care regimen used by contact lens wearers can affect the type and rate of tear deposition on lenses during wear [6,12,33].

In recent years, silicone hydrogel contact lenses have become increasingly popular due to their increased oxygen permeability, resulting in decreased hypoxic-related problems [34]. Silicone hydrogel contact lenses are now commonly used on a daily wear (DW) modality. The DW schedule requires an effective and efficient lens care product to remove deposits from contact lenses and kill adhered bacteria. Multipurpose disinfecting solutions (MPDSs) are the most popular type of lens care product used with silicone hydrogel contact lenses [35]. MPDSs contain antimicrobial components (such as polyquarterium-1 [polyquad], myristamidopopyl dimethylamine [Aldox], or polyhexamethylene biguanide [PHMB]) along with buffer systems, surfactants, and preservatives [36]. These components, however, may absorb to the contact lens surface and change the rate or type of tear deposition. Hydrogel lenses used with a polyquad/citrate-based system have been shown to adsorb less protein compared with lenses used with a PHMB/poloxamine-based solution [37]. Higher amounts of cholesterol oleate could be extracted from senofilcon A contact lenses after use with a lens care system preserved with polyquad and myristamidopopyl dimethylamine and containing poloxamine as a surfactant than a peroxide-based system containing Pluronic 17R4 as a surfactant [38]. Care solutions containing PHMB may also be associated with increased corneal and conjunctival staining compared with the products containing polyquad [39,40]. Zhao et al. [41] have reported correlations between the amount of total protein or cholesterol extracted from contact lenses and the production of solution-induced corneal staining.

Evaluation and quantification of individual proteins in deposits are required to obtain a better understanding of the ability of an MPDS to remove proteins and to examine any relationships with the clinical performance of lenses. The aim of this study was to quantify the amount of lysozyme, lactoferrin, lipocalin-1, proline-rich protein-4, and keratin-1 deposited on silicone hydrogel lenses after wear using multiple-reaction-monitoring (MRM) mass spectrometry (MS) in conjunction with stable isotope–labeled peptide standards. This method has been applied due to its sensitivity, accuracy, and efficiency, and can quantify multiple proteins in a single lens extract [42]. We then examined the impact of multipurpose care solutions on protein deposition and correlations with the clinical performance of the lenses.

## Methods

### Reagents

Trypsin was purchased from Promega (sequencing grade, Madison, WI). All other chemicals were purchased from Sigma Aldrich (St. Louis, MO) were high-performance liquid chromatography grade, as was water.

### Worn contact lens samples and subjective responses

The studies from which worn contact lenses were obtained complied with the Declaration of Helsinki, and approval was obtained from a local human research ethics committee. Before enrolling, participants signed an informed consent form. Studies were registered on a clinical trials website (Australian New Zealand Clinical Trials Registry, ANZCTR, No. ACTRN12609000165280, ACTRN12609000640202, and ACTRN12610001068055).

Balafilcon A (n=20, Bausch + Lomb, Rochester, NY) contact lenses used with RevitaLens OcuTec (Abbott Medical Optics, Santa Ana, CA; containing PQ-1 and alexidine dihydrochloride as disinfectants and Tetronic 904 as a surfactant) and Biotrue (Bausch + Lomb; containing PQ-1 and PHMB as disinfectants, and poloxamine and sulfobetaine as surfactants) or senofilcon A (n=15, Johnson and Johnson Vision Care, Jacksonville, FL) contact lenses used with RevitaLens OcuTec solutions were collected from a group of 35 participants after wear. Data were compared to that obtained from balafilcon A or senofilcon A contact lenses used with AQuify (CIBA Vision Inc., Atlanta, GA) [42]. Senofilcon A or balafilcon A lenses were worn on a DW schedule for 2 weeks or a month, respectively. The frequency of subjective ocular symptoms, including dryness, blurred vision, lens awareness, and ocular discomfort, was obtained via a questionnaire. Symptoms were scored on a 0–4 scale with 0=do not have this symptom (none), 1=seldom notice this symptom (trace), 2=sometimes notice this symptom (mild), 3=frequently notice this symptom (moderate), and 4=always notice this symptom (severe). Investigators wearing latex gloves removed contact lenses from each participant. Lenses were then rinsed in 2 mL sterile PBS (1.15 g Na_2_HPO_4_, 0.2 g KH_2_PO_4_, 0.2 g KCl, 8 g NaCl, in 1 liter distilled H_2_O, pH 7.4) and kept dry in −80 °C before the deposits were evaluated.

### Protein extraction and enzymatic treatment of worn contact lenses

A technique reported by Omali et al. [42] was used for extracting worn lenses. Briefly, contact lenses were extracted in 250 µl of a 50:50 mixture of 0.2% (vol/vol) trifluoroacetic acid and acetonitrile [43], in the dark at ambient temperature for 24 h. Contact lens extracts were evaporated to dryness using a SpeedVac (SC110A, Savant, Thermo) and then digested with trypsin at 37 °C for 18 h. Unworn lenses washed in PBS or MPDS were used as control for extraction and enzymatic treatment.

### Liquid chromatography multiple-reaction-monitoring mass spectrometry analysis of trypsinized lens extracts

The proteins lysozyme, lactoferrin, lipocalin-1, proline-rich protein-4, and keratin-1 were selected to be quantified as they have been isolated previously from contact lens deposits [11-13]. As described by Omali et al. [42], liquid chromatography (LC)–MS-MS analyses were performed using a fritless nano column manufactured according to Gatlin et al. [44] LC–MRM-MS analyses were performed on a 4000 QTRAP hybrid triple quadrupole linear ion trap mass spectrometer (Applied Biosystem/MDS Sciex, Foster City, CA) interfaced with a nanospray ion source operating in positive ion mode and controlled by Analyst 1.5 software (Applied Biosystems). Samples were analyzed with an ion spray voltage of 2.4 kV, curtain gas flow of 12 µl/min, and nebulizing gas flow of 5 µl/min. Peptides were concentrated and desalted onto a micro C18 precolumn (500 µm x 2 mm, Michrom Bioresources, Auburn, CA) with H_2_O:CH_3_CN (98:2, 0.05% TFA) at 15 μl/min. After 4 min of washing, the precolumn was automatically switched (Valco 10 port valve, Houston, TX) into line with a fritless nano column manufactured according to Gatlin [44]. The precolumn was connected via a fused silica capillary (25 cm, 25 µ) to a low-volume tee (Upchurch Scientific, Oak Harbor, WA) and introduced into the 4000 QTRAP mass spectrometer. Peptides were eluted using a linear gradient of H_2_O:CH_3_CN (98:2, 0.1% formic acid) to H_2_O:CH_3_CN (36:64, 0.1% formic acid) at about 300 nl/min over 30 min.

**Figure 1:**
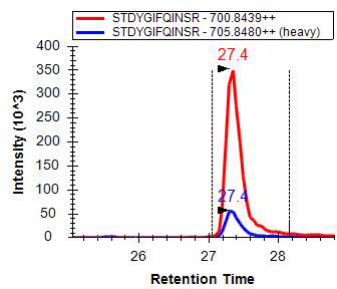
A representative liquid chromatography multiple-reaction-monitoring mass spectrometry extract ion chromatogram of a selected peptide from a lysozyme protein.

After two of the most abundant peptides were selected according to the description given by Omali et al. [42], for each target protein, stock solution of synthesized heavy (with isotope labeling, ^13^C/^15^N) or light (without isotope labeling, ^12^C/^14^N) peptides from Sigma Aldrich were prepared in a water/acetonitrile (vol/vol) and 0.1% formic acid (vol/vol) mixture to achieve the final concentrations of 10 pmol/μl and 1 nmol/μl for heavy and light peptides, respectively. A summary of proteins and MRM transitions monitored in this study is shown in Table 1. A representative LC-MRM/MS extract ion chromatogram of a selected peptide from a lysozyme protein is shown in Figure 1.

**Table 1:** Summary of proteins and MRM transitions.

Protein	Peptides	Precursor ion (m/z)	Product ion (m/z)	*MM
Lysozyme	ATNYNAGDR	491.2	809.3, 695.3, 532.2	980
	ATNYNAGD[RC_6_^13^N_4_^15^]	496.2	819.3, 705.3, 542.2	991
	STDYGIFQINSR	700.8	934.5, 877.4, 764.4	1195
	STDYGIFQINS[RC_6_^13^N_4_^15^]	705.8	944.5, 887.4, 774.4	1203
Lactoferrin	DGAGDVAFIR	510.7	777.4, 720.4, 605.3	1020
	DGAGDVAFI[RC_6_^13^N_4_^15^]	515.7	787.4, 730.4, 615.3	1030
	FQLFGSPSGQK	598.3	920.4, 807.3, 660.3	1195
	FQLFGSPSGQ[RC_6_^13^N_4_^15^]	602.3	928.4, 815.4, 668.3	1203
Lipocalin-1	NNLEALEDFEK	661.3	851.4, 780.3, 667.3	1320
	NNLEALEDFE[RC_6_^13^N_4_^15^]	665.3	859.4, 788.3, 675.3	1329
	GLSTESILIPR	593.3	928.5, 827.4, 698.4	1185
	GLSTESILIP[RC_6_^13^N_4_^15^]	598.3	938.5, 837.5, 708.4	1195
Proline rich protein-4	QLSLPR	357.2	585.3, 472.2, 385.2	713
	QLSLP[RC_6_^13^N_4_^15^]	362.2	595.3, 482.2, 395.2	723
	FPSVSLQEASSFFR	801.4	971.4, 843.3, 714.3	1603
	FPSVSLQEASSFF[RC_6_^13^N_4_^15^]	806.4	981.4, 853.4, 724.3	1611
Keratin-1	SLNNQFASFIDK	692.3	827.4, 680.3, 609.3	1380
	SLNNQFASFID[RC_6_^13^N_4_^15^]	696.3	835.4, 688.3, 617.3	1391
	YEELQITAGR	590.3	887.4, 758.4, 645.3	1178
	YEELQITAG[RC_6_^13^N_4_^15^]	595.3	897.5, 768.4, 655.3	1189

*Molecular mass

### Determination of the amount of protein extracted from worn silicone hydrogel contact lenses

The amount of protein extracted from worn silicone hydrogel contact lenses was determined using a method reported by Omali et al. [42]. Briefly, a standard curve using the peak area ratio of heavy/light peptide (varying amount of light peptides [0 to 4,000 fmol/µl] spiked with heavy peptides to a final concentration of 250 fmol/µl) was created for quantitative analysis. Heavy standard peptides were added to the lens digest samples post-digestion. For each group of lens/MPDS combination, protein deposits were measured on three occasions, and unworn lenses were used as controls on each occasion for any effect of lens components on MRM data.

### Total protein deposition measurement

Following protein extraction from individual worn lenses as described above, total protein measurement for each group of worn contact lens/MPDS combination (n=15 for each) was performed using the LavaPep protein quantification kit (Fluorotechnics, Gladesville, Australia) [45] according to the manufacturer’s instructions. The amounts of total protein deposited on balafilcon A and senofilcon A worn for 14 days in conjunction with RevitaLens OcuTec were similarly determined. Unworn lenses treated in PBS or MPDS were used as control.

### Data analysis

Skyline software [46] was used for data analysis. Pilot experiments indicated that for each group of lens/MPDS combination, a minimum of 14 lenses was needed to show an ex vivo difference of 0.1±0.09 log unit transformed from µg/lens data with a 95% confidence interval and 80% power, and 20 worn balafilcon A and 15 senofilcon A lenses were examined in each group. Data were log transformed before analysis. The linear mixed model (general linear model with random factors) was used to analyze the differences in protein deposited on each lens type ex vivo. Subject number was factored as random intercepts in the linear mixed model to account for within sample correlation.

The correlation between subjective ocular clinical symptoms and specific protein deposition was also investigated using the linear mixed model. For this analysis, subjective ocular symptoms were combined into two categories, none to trace and mild to moderate. There were no cases of severe symptoms. The variance of proteins was compared between the subjects using Levene’s test. An independent sample Student *t* test was used to analyze the differences between total protein deposition within each lens/MPDS type combination. SPSS program 18 was used for all analyses, and p<0.05 was considered significant.

## Results

### Determination of the amount of individual proteins extracted from worn silicone hydrogel contact lenses

The amount of lysozyme, lactoferrin, lipocalin-1, proline rich protein-4, or keratin-1 from worn balafilcon A or senofilcon A lens extracts is shown in Table 2. The amount of protein extracted from contact lenses was affected by the lens material. The amount of lysozyme, lactoferrin, lipocalin-1, and proline rich protein-4 extracted from the balafilcon A lenses (5.08±4.3, 0.12±0.05, 0.49±0.4, and 0.12±0.06 µg/lens, respectively) were significantly higher (p<0.05) than that extracted from the senofilcon A lenses (0.35±0.1, 0.05±0.02, 0.02±0.02, and 0.03±0.03 µg/lens, respectively) when RevitaLens OcuTec was used. Between the two lens types, there was no difference in the amounts of keratin-1 (0.06±0.06 versus 0.05±0.04 µg/lens; p=0.57) when RevitaLens OcuTec was used.

**Table 2:** The amount of specific protein (µg/lens)* extracted from worn balafilcon A and senofilcon A lenses.

**Protein type**	**^£^Balafilcon A AQuify [% of total protein]**	**^£^Senofilcon A AQuify [% of total protein]**	**Balafilcon A RevitaLens OcuTec [% of total protein]**	**Senofilcon A RevitaLens OcuTec [% of total protein]**	**Balafilcon A Biotrue [% of total protein]**
Lysozyme	12.9±9.01 [93.5]	0.88±0.13 [19.5]	5.08±4.30 [49.8]	0.35±0.15 [20.6]	3.4±2.52 [30.9]
Lactoferrin	0.84±0.50 [6.1]	0.50±0.10 [11.1]	0.12±0.05 [1.2]	0.05±0.02 [3.0]	0.05±0.01 [0.45]
Lipocalin-1	2.06±1.64 [14.9]	0.27±0.23 [6.0]	0.49±0.44 [4.9]	0.02±0.02 [1.2]	0.41±0.15 [3.7]
Proline rich protein-4	0.11±0.04 [0.8]	0.15±0.12 [3.3]	0.12±0.06 [1.2]	0.03±0.03 [2.0]	0.12±0.04 [1.1]
Keratin-1	0.83±0.61 [6.1]	0.77±0.20 [17.16]	0.06±0.06 [0.6]	0.05±0.04 [3.0]	0.5±0.94 [4.6]
**Total protein	121%	67%	58%	30%	41%

* Data are shown as mean ± standard deviation; **Percentage of total protein was achieved by adding up the amount of all proteins tested in this study; ^£^Data from balafilcon A or senofilcon A in combination with AQuify have been reproduced from a report by Omali et al [42].

A comparison can be made of the data related the amounts of protein deposited on balafilcon A and senofilcon A when used with AQuify [42] with the protein deposit data for balafilcon A and senofilcon A/RevitaLens OcuTec and balafilcon A/Biotrue combinations to have a better understanding of the effect of care solutions on the rate of protein deposition on lenses. Within each lens type, the amount of protein extracted was affected by the type of care system used. More protein was extracted from both lens types when they were used with AQuify than with RevitaLens OcuTec (p<0.05), and this remained the case for balafilcon A lenses when used with AQuify compared to Biotrue (p<0.05). When the balafilcon A lens was used with Biotrue or RevitaLens OcuTec, there were no significant differences in the amount of target proteins extracted (p>0.05). The amount of proline rich protein-4 extracted from balafilcon A (0.11±0.04, 0.12±0.06, or 0.12±0.04) was not affected when used with AQuify, RevitaLens OcuTec, or Biotrue, respectively (p>0.05).

### Total protein quantification of contact lens extracts

The total proteins extracted from the balafilcon A or senofilcon A lenses used with AQuify or RevitaLens OcuTec and balafilcon A used with Biotrue lens extracts are shown in Table 3. The amount of total protein extracted from the balafilcon A/AQuify combination (13.8±1.9 µg/lens) was significantly (p=0.0001) higher than that extracted from balafilcon A used with RevitaLens OcuTec (10.2±4.1 µg/lens). This was also the case when senofilcon A lenses were used with AQuify versus RevitaLens OcuTec (3.8±1.5 versus 1.7±0.6 µg/lens, respectively; p=0.01). However, the amount of total protein deposited on balafilcon A lenses used with Biotrue (11.5±3.1) was similar to that used with RevitaLens OcuTec (10.2±4.1 µg/lens, p=0.08). Balafilcon A and senofilcon A lenses in conjunction with the use of RevitaLens OcuTec worn for 14 days deposited 9±2.12 and 2±1.03 µg/lens of total protein, respectively. The combination of lysozyme/lactoferrin/lipocalin-1/proline rich protein-4 and keratin-1 as a proportion of the total protein was 121% for balafilcon A/AQuify, 67% for senofilcon A/AQuify, 58% for balafilcon A/RevitaLens OcuTec, 30% for senofilcon A/RevitaLens OcuTec, and 41% for balafilcon A/Biotrue MPDS (Table 3).

**Table 3:** Total protein deposition on worn contact lenses.

Lens Type	*MPDS Type	**Total Protein Deposition (µg/lens)
Balafilcon A	AQuify	13.8±1.9
	RevitaLens OcuTec	10.2±4.1
	Biotrue	11.5±3.1
Senofilcon A	AQuify	3.8±1.5
	RevitaLens OcuTec	1.7±0.6

*Multipurpose disinfecting solution; **Data are shown as mean ± standard deviation

**Figure 2:**
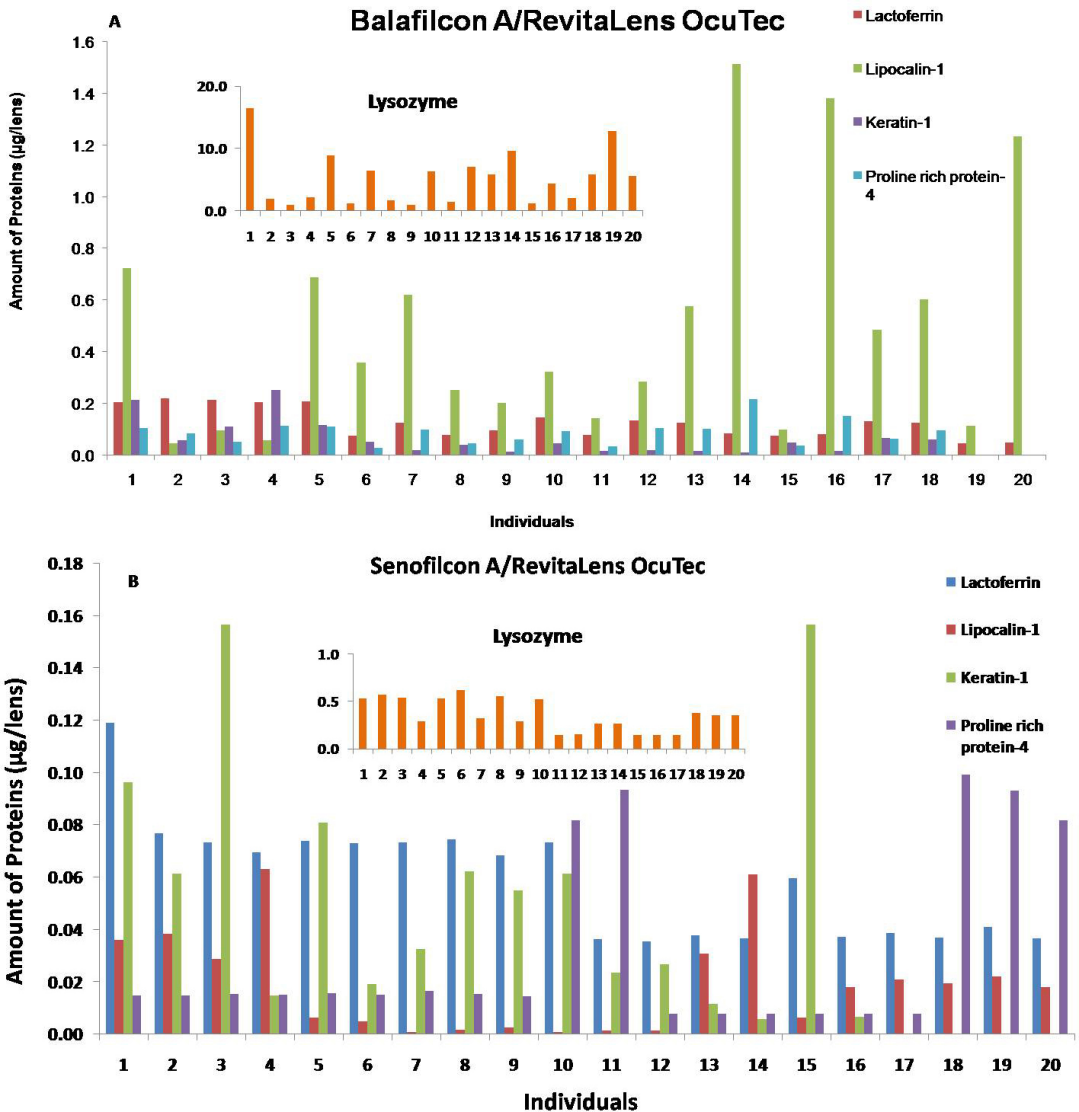
The variability of individual proteins detected in balafilcon A (**A**) or senofilcon A (**B**) lens materials when used with RevitaLens OcuTec.

The amount of specific proteins in the lens extracts was compared for the individual wearers (Figure 2A,B). The variability in the amount of lysozyme extracted from the balafilcon A or senofilcon A lenses in combination with RevitaLens OcuTec was significantly (p=0.0001) higher than other proteins tested in this study with a maximum extracted of 16.4 or 0.61 µg/lens, respectively, for one person and a minimum extracted of 0.90 or 0.14 µg/lens, respectively, for another person. Except for keratin-1 (p>0.7), the variability of the tested proteins in this study with balafilcon A was significantly (p<0.005) higher than that with senofilcon A.

### Correlation between specific protein deposition and clinical symptoms

An analysis of the amount of each individual protein extracted from worn lenses with subjective symptoms showed that patients who reported mild to moderate symptoms of dryness had greater levels of keratin-1 (0.6±0.7 versus 0.3±0.5, p=0.03) and lipocalin-1 (1.2±1.6 versus 0.5±0.7, p=0.01) deposited on their lenses compared to those who reported none to trace symptoms of dryness. There was no correlation between the amount of any protein extracted from lenses and symptoms of blurred vision, lens awareness, and discomfort.

## Discussion

This is the first study to report quantification of different proteins extracted from a single worn contact lens in addition to the impact of the MPDSs on individual protein deposits. Lysozyme, lactoferrin, lipocalin-1, keratin-1, and proline rich protein-4 were selected to be quantified in this study as they have been frequently detected, although not all previously quantified, in contact lens extracts [1,11-13]. Irrespective of MPDS use, lysozyme was detected as the most abundant protein in all contact lens extracts. This finding is consistent with previous reports [47,48]. Similar to the report by Omali et al. [42] where higher amounts of individual proteins were extracted from balafilcon A compared to senofilcon A when the lenses were used with AQuify, the current study shows RevitaLens OcuTec higher levels of proteins extracted from balafilcon A than senofilcon A lens materials. Differences in the lens materials, ionicity [47,49,50], and microporous structure [51] of the balafilcon A lenses could contribute to this finding.

The differences in extractable protein from lenses when used with different MPDSs might be due to several factors, including the disinfectant and surfactant in the MPDS. Lenses that had been used with MPDS containing either PQ-1/alexidine dihydrochloride and Tetronic 904 (RevitaLens OcuTec) or PQ-1/PHMB and poloxamine/sulfobetaine (Biotrue) had fewer proteins (total or specific) that could be extracted compared with those used with the PHMB and poloxamer 407–based MPDSs. Emch et al. [1] found that OPTI-FREE Express (containing PQ-1/myristamidopropyl dimethylamine [MAPDA] and poloxamine) was more efficient in removing proteins from lotrafilcon B or galyfilcon A (a base polymer similar to senofilcon A) silicone hydrogel lenses compared to AQuify. Senchyna et al. [52] compared lysozyme deposition and its activity on worn etafilcon A hydrogel and balafilcon A silicone hydrogel contact lenses and reported that when these lenses were used with a care solution that was preserved with PHMB, lysozyme deposition was greater on etaﬁlcon A lenses; however, using either solution did not affect the amount of lysozyme deposition on balafilcon A lenses. The authors also indicated that when a PHMB/poloxamine-based system was used, lysozyme was denatured to a greater degree compared with the use of a PQ-1/poloxamine-based system [52]. This indicates protein deposition on silicone hydrogel lenses is influenced by several excipients in MPDS formulations as well as the chemistry of the lenses themselves.

Following deposition on the lens surface, lysozyme may become denatured, and previous reports have attributed this to clinical complications such as immunological responses of ocular surface [5,53]. However, Wright et al. [54] examined Biotrue care solution containing protein-stabilizing agents (hyaluronic acid, poloxamine, and sulfobetaine 10) and concluded that this care solution prevents the denaturation of physiologic levels of human lactoferrin and lysozyme and that stabilized proteins retain their function. Given that Omali et al. [42] indicated that efficiency of protein extraction from the balafilcon A or senofilcon A lens materials was not 100%, it is possible that other, as yet unidentified, proteins may also be contributing to immunological responses and that the cleaning solution/procedure is in part responsible for their presence.

Zhao et al. [6] found that greater amounts of total protein could be extracted from balafilcon A compared with senofilcon A lenses, which is in agreement with the current finding. Pucker et al. [55] reported that 3.0±1.9 µg/lens total protein could be extracted from galyfilcon A lens material when used with AQuify. Although our study did not assess galyfilcon A lenses, senofilcon A and galyfilcon A are similar materials, with both composed of monofunctional polydimethylsiloxane, N,N-dimethylacrylamide, poly (2-hydroxyethyl methacrylate), siloxane macromer, and poly (vinyl pyrrolidone). Our finding of 3.8±1.5 µg/lens total protein extracted from senofilcon A when used with AQuify is therefore in agreement with Pucker et al.’s findings [55]. Zhao et al. [6] extracted only 1.1±2.8 µg/lens from galyfilcon A when used with AQuify and 0.7±0.5 µg/lens from senofilcon A when used with AQuify, and these differences are likely to be due to the use of different extraction procedures (4 M urea, 0.01% sodium dodecylsulfate, and 1 mM dithiothreitol, pH 7.4 by Zhao et al. compared to a 50:50 mixture of 0.2% (vol/vol) trifluoroacetic acid and acetonitrile in the current investigation).

The higher protein deposition on balafilcon A lenses might also have been influenced by the replacement schedule compared to senofilcon A (1 month versus 2 weeks). Although Zhao et al. [6] quantified total amounts of protein from balafilcon A and senofilcon A lenses that were daily worn for 30 days and used with OPTI-FREE Express (PQ-1/MAPDA based) and indicated that higher levels of protein could be quantified from balafilcon A (23.1±5.8 µg/lens) lenses compared with senofilcon A (0.1±0.1 µg/lens), protein denaturation and deposition on contact lens materials are not necessarily equivalent for a 1 month versus 2-week replacement schedule.

In the current study, participants were advised to use RevitaLens OcuTec and Biotrue as rub and rinse solutions whereas AQuify was used as a rinse-only solution [42]. Rubbing could change the binding/denaturation of proteins to contact lens material. Mok et al. [56] demonstrated the importance of the rub step to remove proteins efficiently from worn lenses. Cho et al. [57] reported that rubbing lenses during the cleaning procedure is an effective way of removing deposits. Using a rub and rinse regime has also been shown to improve the disinfection efficacy of MPDSs when used with silicone hydrogel and conventional lenses by Zhu et al. [58]. Peterson et al. [59] reported reduced levels of corneal staining induced after 2 h of lens wear with the combination of balafilcon A and PHMB-based lens care solution when a rub and rinse step was included in the cleaning procedure before overnight soaking.

When balafilcon A lenses were used with AQuify, the combined amount of lysozyme, lactoferrin, lipocalin-1, proline rich protein-4, and keratin-1 was approximately equivalent to the amount of total protein extracted from lenses, indicating that these were indeed the major proteins extracted from this lens/MPDS combination. However, the percentage of these specific proteins to total protein exceeded 100%, and this could be because the amount of specific and total protein on lenses was determined and compared using different methods, MRM and LavaPep total protein assay, respectively, as well as the effects of averaging amounts from different lenses. In addition, with other lens/MPDS combinations these proteins contributed between 30% and 67% of total protein. This implies that there may be other proteins deposited on the lens surface that could make up to 70% of the total proteins. These proteins might include secretory immunoglobulin A, heparan sulfate proteoglycan, other keratins (e.g., keratin 10), albumin, or apolipoprotein J, which have been identified by mass spectrometry in lens extracts [11]. Furthermore, de Souza et al. [60] used mass spectrometry and showed that a large number of proteins (491) are present in tears, and it is possible that some of these also deposit on lenses during wear. Omali et al. [42] reported the protein recoveries of lysozyme, lactoferrin, lipocalin-1, and keratin-1 from the balafilcon A or senofilcon A lens materials were ≥64% or 67%, respectively, using the MRM assay and ≥79% or 65%, respectively, using the LavaPep assay, indicating that the majority of protein could be removed during extraction. However, there is the possibility that the protein retained on the contact lens materials following the extraction procedure could still be involved in contact lens–induced symptoms. The identity and quantity of other protein types deposited on lenses used with different MPDS should be investigated, and the use of the MRM/MS technique is appropriate.

A post hoc analysis showed that increased dryness symptoms correlated with increased amounts of keratin or lipocalin deposited on contact lenses. Intolerant contact lens wearers (those who discontinue lens wear due to ocular discomfort symptoms) have been shown to have significantly higher amounts of lipocalin in their tears [61]. Perhaps, due to increased amount of lipocalin in the tears of intolerant contact lens wearers, higher levels of lipocalin could accumulate on their lenses during wear. Keratin expression is often tissue specific, with expression of keratin-1, the keratin examined in these studies, limited to the epidermidis of the skin [62-66]. Keratins associated with the cornea or conjunctiva are keratin 3/12 and 4/13, respectively [62,67-69]. Thus, it is likely that keratin-1 on the surface of lenses comes about due to lens handling. Although the findings for this study point to an association of symptoms and increased deposition of certain proteins, this study was not designed specifically to investigate this hypothesis, and further investigation of the correlation between specific protein deposition and clinical symptoms is warranted.

Overall, this study shows that lens/MPDS combinations affect the levels of specific proteins that can be extracted from lenses after wear. Lower amounts of proteins were extracted from lenses when used with PQ-1-based systems or PQ-1/PHMB combination regardless of the surfactant used, compared to a PHMB/Poloxamer 407 MPDS. This result could have been influenced by rubbing and rinsing of lenses with the two PQ-1-based systems compared to rinse-only with the PHMB-based MPDS.
